# Microstructure and Properties of Micro/Nano-Scale (TiB_2_ + TiC)/Al Composites Prepared by Ti-B_4_C Reactive Sintering and Spark Plasma Sintering

**DOI:** 10.3390/ma19071449

**Published:** 2026-04-04

**Authors:** Wenchao Huang, Dongting Li, Renquan Wang, Ying Liu

**Affiliations:** College of Materials Science and Engineering, Sichuan University, Chengdu 610065, China

**Keywords:** (TiB_2_ + TiC)/Al composites, micro/nano-scale, mechanical properties, microstructure, tribological properties

## Abstract

In this work, micro/nano-scale (TiB_2_ + TiC)/Al composites with reinforcement contents ranging from 0 to 30 wt.% were fabricated by the combination of Ti-B_4_C reactive sintering and spark plasma sintering (SPS). The results indicate that a sintering temperature of 1400 °C is essential for achieving a complete reaction between Ti and B_4_C, successfully producing a bimodal TiB_2_-TiC reinforcement consisting of nano-scale and micro-scale particles. Microstructure analysis reveals that the addition of micro/nano-scale TiB_2_ and TiC ceramic particles significantly refines the grain size of the Al matrix from 11.52 μm in pure Al to 1.09 μm in the 30 wt.% (TiB_2_ + TiC)/Al composite. As the TiB_2_ and TiC contents increase, Vickers hardness and compressive yield strength increase progressively, while the uniform compressive plastic strain first increases and then decreases. The 20 wt.% (TiB_2_ + TiC)/Al composite demonstrates the optimal comprehensive properties, with a compressive yield strength of 196.4 ± 6.1 MPa, an ultimate strength of 914.6 ± 20.1 MPa, and a uniform plastic strain of ~73.2%, as well as minimal wear rates of (3.143 ± 0.194) × 10^−4^ mm^3^/(N·m), 1.676 ± 0.251× 10^−3^ mm^3^/(N·m) and (3.093 ± 0.335) × 10^−3^ mm^3^/(N·m) at 1 N, 3 N, and 5 N, respectively. This improvement stems from the combined effects of grain refinement, dispersion strengthening, enhanced load-bearing capacity and reduced adhesive wear via the TiB_2_ and TiC reinforcements.

## 1. Introduction

Owing to the advantageous such as low density, high specific strength, good formability, etc., particle-reinforced Al matrix composites (PRAMCs) are extensively utilized in transportation, aerospace, and electronics [1,2,3,4]. Various ceramic particles, such as TiB_2_ [1,2,3,4,5,6], Al_2_O_3_ [7,8], SiC [2,8,9], B_4_C [8,10] and TiC [6,11,12], have been employed to prepare Al matrix composites. Among them, TiC and TiB_2_ are considered as ideal reinforcements for preparing high-performance Al matrix composites due to their high melting point, exceptional hardness and modulus, good wettability and excellent chemical stability with the Al matrix [4,6,11,13]. These advantages can not only refine the Al matrix but also transfer loads and impede dislocation, thus significantly improving the mechanical performance of Al and its alloys. Consequently, (TiB_2_ + TiC)/Al composites have been the focus of significant research interest in recent decades.

To fabricate high-performance (TiB_2_ + TiC)/Al composites, several techniques have been utilized, such as powder metallurgy, stir casting, reactive sintering and self-propagating high-temperature synthesis (SHS). Among them, SHS as an efficient material fabrication technology and has many advantages such as being economical, convenient and rapid, etc., making it very suitable for fabricating high-performance Al matrix composites. Moreover, a key advantage of SHS is its in situ synthesis of reinforcements within the Al matrix, which results in fine particle size, inherent thermal stability and strong interfacial bonding, collectively contributing to marked improvements in mechanical performance. Li et al. [14] prepared (TiC + TiB_2_)/Al composites via SHS and hot-pressing, achieving good mechanical properties. Zhong et al. [15] prepared TiB_2p_-TiC_p_-reinforced Al matrix composites by SHS, which exhibited superior high-temperature strength and wear resistance. Yi et al. [16] reported a significantly enhanced microhardness and elastic modulus in (TiB_2_ + TiC)/AlSi10Mg composites prepared via selective laser melting and SHS. Yang et al. [17] demonstrated that the strong interface bonding in high-volume-fraction (TiC + TiB_2_)/Al composites is critical to achieving substantial increases in compressive yield strength, ultimate compressive strength and plastic strain. However, this method still faces challenges such as agglomeration, inhomogeneity and clustering of the reinforcement, which adversely affect the mechanical properties of Al matrix composites, thereby limiting their broader application in demanding sectors such as aerospace and transportation [4,18,19].

In recent years, nano-scale and multi-scale hybrid reinforcement have shown great potential for enhancing the mechanical properties of Al matrix composites. Adding nano- and micro-sized ceramic particles can produce a synergistic strengthening effect (e.g., Orowan strengthening, grain refinement and dispersion strengthening), thereby achieving a simultaneous improvement in strength and ductility [20,21,22]. Cheng et al. [23] developed multi-scale (TiB_2_ + TiC)/Al layered composites, where micro/sub-micro TiC particles provided a supportive effect, leading to exceptional strength, toughness and wear resistance. Zheng et al. [24] prepared micro/nano-TiB_2_-reinforced 6061 Al matrix composites via high-energy ball milling, inert-atmosphere sintering and hot-pressing, demonstrating that micro/nano TiB_2_ can markedly improve mechanical properties and wear resistance. However, there are still few reports on multi-scale (TiB_2_ + TiC)/Al matrix composites. Furthermore, SPS as an advanced consolidation technique has many advantages including short sintering time, low sintering temperature, high efficiency and controllable microstructure [6,25], making it very suitable for preparing high-performance (TiC + TiB_2_)/Al composites. Thus, micro/nano-scale (TiC + TiB_2_)/Al composites were prepared by Ti-B_4_C reactive sintering and SPS, and the influence of TiC and TiB_2_ content on the microstructure, mechanical and tribological performance of the (TiC + TiB_2_)/Al composites was investigated.

## 2. Materials and Methods

The micro/nano-scale (TiC + TiB_2_)/Al composites with varying TiC and TiB_2_ contents (0, 5, 10, 15, 20, 25 and 30 wt.%, respectively) were prepared by Ti-B_4_C reactive sintering and SPS, using pure Al powders (99.99 wt.%, about 45 μm; Zhongchun New Materials Technology Co., Ltd., Beijing, China), pure Ti powders (99.5 wt.%, about 10 μm; Beijing xingrongyuan Technology Co., Ltd., Beijing, China) and B_4_C powders (99.9 wt.%, about 1.0 μm; ZhenHan New Materials Co., Ltd., SuZhou, China). Firstly, pure Ti and pure B_4_C powders were weighed according to the reaction 3Ti + B_4_C = TiC + 2TiB_2_, and then the Ti and B_4_C powders were mixed in a 3D mixing machine (JXHY-2L, Shanghai Jingxin Industrial Development Co., Ltd., Shanghai, China) at 30 rpm for 10 h. After mixing, the mixed powder was then sintered in a tube furnace (GSL-1500X, Hefei Kejing Materials Technology Co., Ltd., Hefei, China) at temperatures of 1100, 1200, 1300 and 1400 °C for 2 h with a heating rate of 10 °C/min under a high-purity argon atmosphere (99.999%) to ensure complete reaction. Secondly, to obtain fine TiC and TiB_2_ ceramic particles, the sintered bulk sample was first ground into powder, followed by high-energy ball milling in a planetary ball mill (YXQM-1L, Changsha Miqi Instrument and Equipment Co., Ltd., Changsha, China) for 2 h under the following conditions: an ethanol medium, zirconia ball-milling jar and balls, a ball-to-material ratio (wt.%) of 10:1 and a rotational speed of 400 rpm. After that, the ball-milled powder was dried in a vacuum oven (DZF-6050T, Guansen Biotechnology Co., Ltd., Shanghai, China) under vacuum conditions at 80 °C for 8 h. Finally, the as-prepared TiC and TiB_2_ and Al powders were blended for 10 h at 15 rpm in a horizontal roller mixer (QM-1.5, Changsha Tianchuang Powder Technology Co., Ltd., Changsha, China) at a ball-to-material ratio (wt.%) of 5:1. After that, the powder blends were sintered into cylindrical samples (Φ20 × 15) by SPS (LABOX-1050, Tokyo, Japan) using a graphite mould (Φ20.4 × 50) at 550 °C for 10 min under 40 MPa and a heating rate of 100 °C/min, followed by furnace cooling to room temperature (RT).

The Archimedes principle was used to determine the relative density of the (TiC + TiB_2_)/Al composites. Vickers hardness was measured at RT using a hardness tester (Via-F, Matsuzawa, Japan) under a 1 kgf load with a 15 s dwell time. Each reported Vickers hardness value is the average of seven measurements per sample. The compressive properties were evaluated at RT on an electronic universal testing machine (C45.105Y, MTS, Shanghai, China) at a compression speed of 0.2 mm/min using cylindrical samples (Φ6 × 9). Triplicate tests were conducted for each sample to ensure reliability. Tribological experiments were conducted using a reciprocating ball-on-disk tribometer (UMT TriboLab, Bruker, Beijing, China) under ambient conditions. An Al_2_O_3_ ball with a 6 mm diameter served as the counterpart. Tribological tests were conducted using a sliding velocity of 10 mm/s, applied loads of 1–5 N, a stroke length of 6 mm, and a total duration of 1800 s. The coefficient of friction (COF) was recorded in real-time, and the reported COF is the average value over the steady-state region (after 900 s) of the friction curve. To ensure reproducibility, each test was repeated three times under controlled ambient conditions with the relative humidity maintained at 60 ± 5% and the RT at 26 ± 2 °C during the testing process. And the Al_2_O_3_ counterface ball was replaced after each frictional experiment. The wear rate (W) was calculated according to the literature using the standard formula: W = V/(F × S) [26]. After the dry sliding tests, the wear volume (V) was measured using a three-dimensional optical profiler (ContourX-200, Bruker, Beijing, China). For accuracy, measurements were taken at a minimum of three different locations along the wear track, and the results were averaged. The tribological test samples were ground using silicon carbide sandpaper with different grits (80–1200) and then mechanical polished with a finer-grained (0.05 μm) silica suspension.

Phase analysis of the (TiC + TiB_2_)/Al composites was performed via X-ray diffraction (XRD, D6 Phaser, Bruker, Berlin, Germany) with Cu Kα radiation (40 kV; 30 mA). Scans were conducted from 20° to 90° (2θ) with a scanning time of 1 s and a step size of 0.02°. The microstructures and elemental distribution of (TiC+TiB_2_)/Al composites were examined by scanning electron microscope (SEM, JSM-7900F, JEOL, Tokyo, Japan) equipped with EDS (Ultim MAX 65, Oxford, London, UK) and transmission electron microscope (TEM, Talos F200X, TMO, Bellevue, WA, USA). The grain size and distribution of the (TiC + TiB_2_)/Al composites were characterized via electron backscatter diffraction (EBSD, GeminiSEM 500, Zeiss, Oberkochen, Germany).

## 3. Results and Discussion

**Figure 1:**
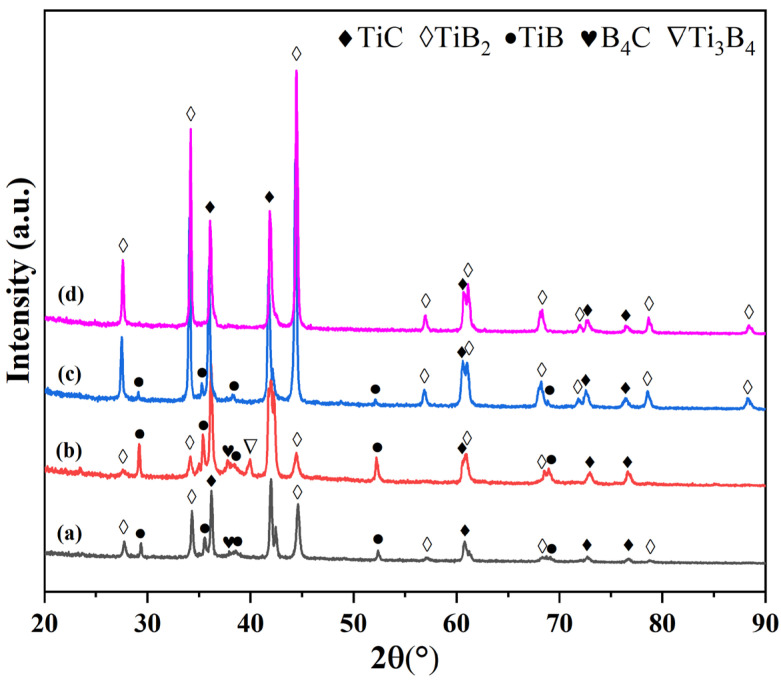
XRD patterns of Ti-B_4_C powders sintered at different temperatures: (**a**) 1100 °C; (**b**) 1200 °C; (**c**) 1300 °C; (**d**) 1400 °C.

Figure 1 shows the XRD patterns of Ti-B_4_C powders sintered from 1100 °C to 1400 °C. With increasing sintering temperature, the diffraction peak intensities of TiC and TiB_2_ progressively increased, indicating a progressively more thorough reaction between Ti and B_4_C. After sintering at 1100 and 1200 °C, the main products were predominantly TiC and a minor amount of TiB_2_, along with traces of the TiB and Ti_3_B_4_ intermediate phases. Moreover, a trace of unreacted B_4_C was also detected, confirming the reaction was incomplete at these temperatures. When sintered at 1300 °C, only a trace of the TiB intermediate phase remained, while the Ti_3_B_4_ intermediate phase had completely disappeared. The final products were predominantly TiC and TiB_2_, indicating that the reaction was nearing completion. After sintering at 1400 °C, all the intermediate phases had completely disappeared, and only TiB_2_ and TiC peaks were detected in the XRD patterns, indicating a complete reaction between Ti and B_4_C. Therefore, high-purity TiB_2_ and TiC ceramic powders can be successfully synthesized via the Ti-B_4_C reaction at 1400 °C.

**Figure 2:**
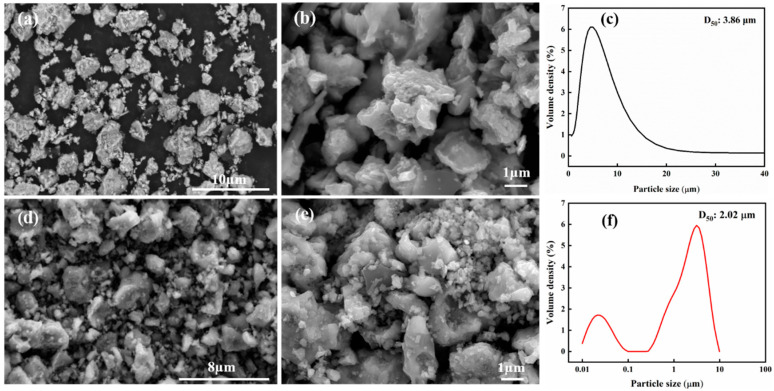
SEM images of TiB_2_ and TiC powders before (**a**,**b**) and after (**d**,**e**) ball milling; particle size distribution of TiB_2_ and TiC powders before (**c**) and after (**f**) ball milling.

SEM images and the particle size distribution of TiB_2_ and TiC powders are displayed in Figure 2 before and after ball milling. As seen in Figure 2a,b, the TiB_2_ and TiC powders synthesized via reactive sintering have a relatively large particle size with a median particle size (D_50_) of about 3.86 µm (Figure 2b), following a Gaussian distribution. Following ball milling, the particle size of the TiB_2_ and TiC powders was significantly reduced, following a bimodal distribution with a median particle size (D_50_) of about 2.02 μm (Figure 2d). In addition, numerous fine particles smaller than 100 nm (Figure 2e) were observed as shown in Figure 2c. Compared with the as-synthesized TiB_2_ and TiC powders, the particle size of the ball-milled TiB_2_ and TiC powders was reduced by nearly 50%. This is mainly attributed to the significant impact and shear forces generated from vigorous collisions between the grinding balls and powders as well as the powder particles themselves during ball milling, thereby causing repeated fracture and fragmentation and ultimately leading to the formation of nanoparticles [27]. Therefore, after ball milling, the TiB_2_ and TiC ceramic powders exhibit a bimodal distribution, consisting of a minority of nanoparticles and a majority of microparticles. The above results indicate that multi-scale TiB_2_ and TiC ceramic powders can be successfully synthesized by Ti-B_4_C reactive sintering.

**Figure 3:**
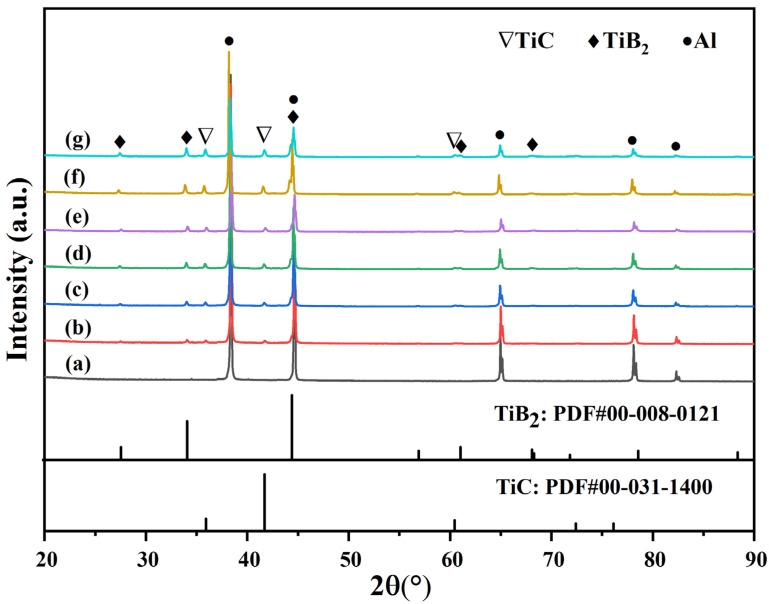
XRD analysis of (TiC + TiB_2_)/Al composites: (**a**) 0 wt.%; (**b**) 5 wt.%; (**c**) 10 wt.%; (**d**) 15 wt.%; (**e**) 20 wt.%; (**f**) 25 wt.%; (**g**) 30 wt.%.

XRD analysis of the micro/nano-scale (TiC + TiB_2_)/Al composites with varying TiC and TiB_2_ content are shown in Figure 3. Compared with pure Al, TiB_2_ and TiC phases were identified in all (TiC + TiB_2_)/Al composites, and their diffraction peak intensities increased gradually with increasing TiB_2_ and TiC content. These results indicated that micro/nano-scale (TiC + TiB_2_)/Al composites can be successfully prepared.

**Figure 4:**
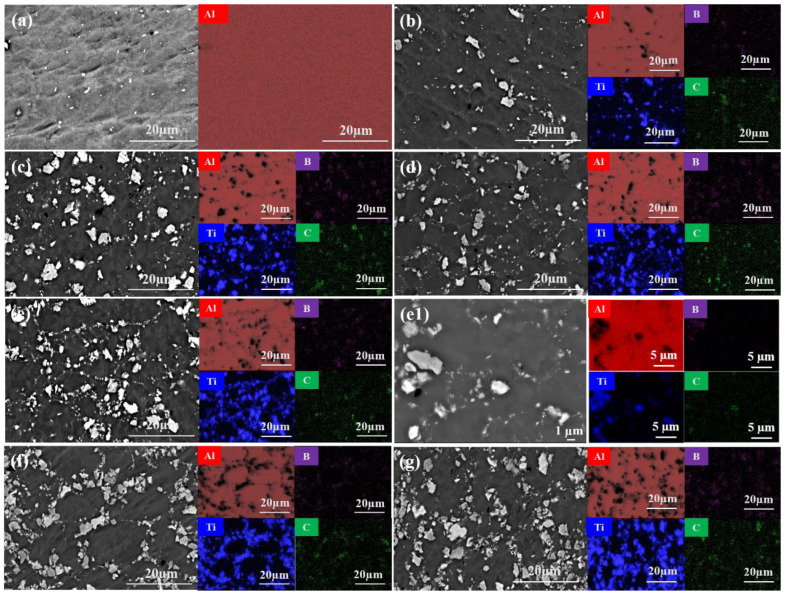
SEM and EDS results of (TiC + TiB_2_)/Al composites: (**a**) 0 wt.%; (**b**) 5 wt.%; (**c**) 10 wt.%; (**d**) 15 wt.%; (**e**,**e1**) 20 wt.%; (**f**) 25 wt.%; (**g**) 30 wt.%.

As shown in Table 1, almost fully dense (TiC + TiB_2_)/Al composites were successfully fabricated, with relative density showing a slight decrease with the increase in TiC and TiB_2_. Figure 4 shows SEM micrographs and corresponding EDS results for the (TiC + TiB_2_)/Al composites with varying TiB_2_ and TiC contents. As shown in Figure 4a, there are no other phases in pure Al. As shown in Figure 4b–e, TiB_2_ and TiC phases are detected in the (TiC + TiB_2_)/Al composites after adding TiB_2_ and TiC ceramic particles. At contents up to 20 wt.%, a uniform distribution of TiB_2_ and TiC ceramic particles is observed in the Al matrix, which effectively hinders grain boundary migration and dislocation motion, thus enhancing strength and hardness and consequently improving the tribological properties by dispersion strengthening. Additionally, the micro/nano-scale TiC and TiB_2_ tend to aggregate at grain boundaries, as shown in the high-magnification image of Figure 4(e1), resulting in grain refinement [28]. However, when the content of TiB_2_ and TiC is increased to 25 wt.% or 30 wt.%, the ceramic particles agglomerate in localized regions, as shown in Figure 4f,g, which can induce severe stress concentration and cause them to act as potential sites for crack initiation, promoting crack initiation and propagation and consequently degrading the mechanical and tribological performance of (TiC + TiB_2_)/Al composites [15].

**Figure 5:**
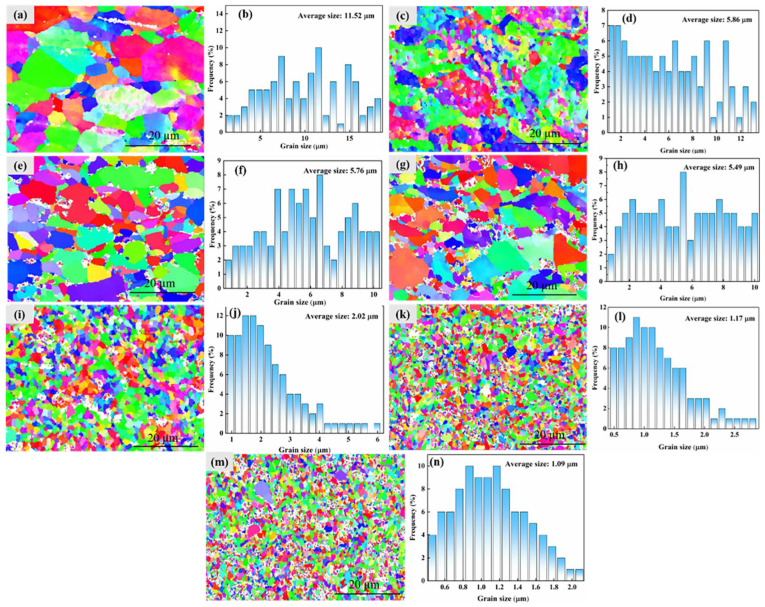
EBSD results of (TiC + TiB_2_)/Al composites: (**a**,**b**) 0 wt.%; (**c**,**d**) 5 wt.%; (**e**,**f**) 10 wt.%; (**g**,**h**) 15 wt.%; (**i**,**j**) 20 wt.%; (**k**,**l**) 25 wt.%; (**m**,**n**) 30 wt.%.

Figure 5 shows an EBSD analysis of (TiC + TiB_2_)/Al composites with varying TiB_2_ and TiC contents. As shown in Figure 5a, the pure Al exhibits a coarse, heterogeneous grain structure with an average grain size of about 11.52 μm. The grain size distribution exhibits the widest range, from 1.54 to 18.16 μm, and follows an approximately log-normal distribution. Adding TiB_2_ and TiC ceramic particles leads to significant grain refinement of the Al matrix. As TiB_2_ and TiC content rises from 5 to 15 wt.%, the average grain size is markedly reduced to about 5.86, 5.76 and 5.49 μm, respectively (Figure 5d,f,h). Accordingly, the grain size distribution range becomes narrower and the grain structure becomes more homogeneous. However, the (TiC + TiB_2_)/Al composites still show a heterogeneous grain structure consisting of coarse and fine grains as shown in Figure 5c,e,g, and their grain size distribution remains an approximately log-normal distribution. Further increasing the TiB_2_ and TiC content to 20, 25 and 30 wt.% results in an average grain size of about 2.02, 1.17, and 1.09 μm, respectively (Figure 5i–n). Notably, the grain size distribution curves are very symmetrical and sharp, with sizes primarily concentrated between 0.5 and 1.5 µm (Figure 5l,n), demonstrating a shift to a typical normal distribution. This finer grain size results in more grain boundaries, which can effectively impede dislocation movement, thereby improving strength and hardness and consequently enhancing the tribological properties [26,28]. However, in Figure 5f,g, particle agglomeration of TiC and TiB_2_ in localized regions results in severe stress concentration, potential sites for crack initiation and propagation, and material delamination, thereby aggravating wear. Moreover, the agglomeration of TiC and TiB_2_ particles leads to an inhomogeneous distribution of hard phases on the worn surface, which results in an uneven plastic deformation and exacerbates ploughing of the Al matrix, consequently leading to severe fatigue wear and brittle spallation and significantly reducing the wear resistance of the (TiC + TiB_2_)/Al composite. Hence, the 25 and 30 wt.% (TiC + TiB_2_)/Al composites exhibit a relatively poor friction performance compared with the 20 wt.% (TiC + TiB_2_)/Al composite.

**Figure 6:**
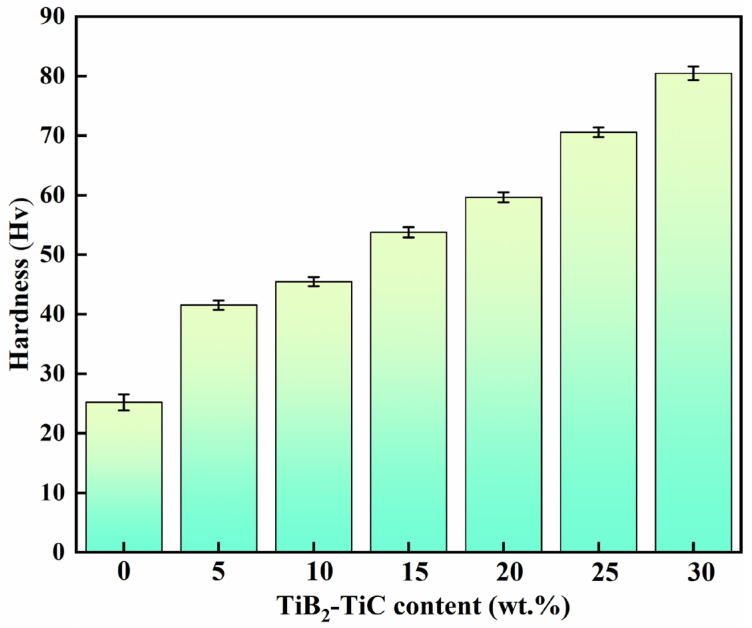
Vickers hardness of (TiC + TiB_2_)/Al composites with varying TiB_2_ and TiC contents.

The Vickers hardness of (TiC + TiB_2_)/Al composites with different TiB_2_ and TiC contents is presented in Figure 6. The incorporation of TiB_2_ and TiC particles leads to a significant enhancement in hardness, which increases progressively with increasing TiB_2_ and TiC content. The average Vickers hardnesses for the (TiC + TiB_2_)/Al composites are 25.2 ± 1.4, 41.5 ± 0.8, 45.4 ± 0.7, 53.8 ± 0.8, 59.6 ± 0.9, 70.6 ± 1.2 and 80.5 ± 1.1 HV_1_, respectively, which are much higher than that for the pure Al matrix. Notably, when the content of TiB_2_ and TiC is 30 wt.%, its Vickers hardness reaches as high as 80.5 ± 1.1 HV_1_, which improves on pure Al by about 220%. The XRD and microstructure results (Figure 3, Figure 4 and Figure 5) show that TiC and TiB_2_ can effectively impede dislocation movement and refine the microstructure of the Al matrix, thereby significantly enhancing Vickers hardness through grain refinement and dispersion strengthening [26,28].

**Figure 7:**
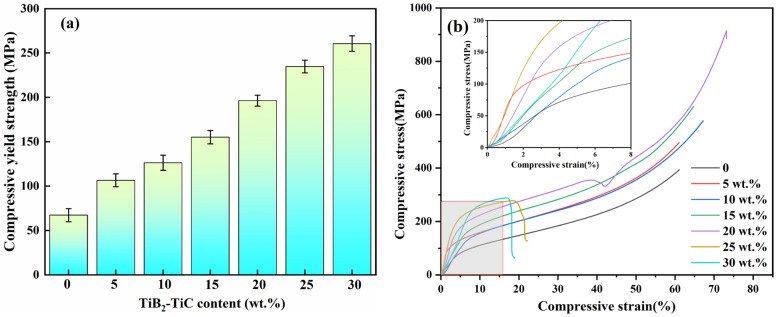
Compressive performance of (TiC + TiB_2_)/Al composites with different TiB_2_ and TiC contents: (**a**) compressive yield strength; (**b**) compressive stress–strain curve.

Figure 7 shows the compressive performance of (TiC + TiB_2_)/Al composites with different TiB_2_ and TiC contents. As shown in Figure 7a, the compressive yield strength of (TiC + TiB_2_)/Al composites increases markedly with the increase in TiB_2_ and TiC, from about 67 ± 7.4 MPa for pure Al to 251 ± 8.7 MPa for the 30 wt.% (TiC + TiB_2_)/Al composites, showing a 275% improvement over pure Al. However, the 30 wt.% (TiC + TiB_2_)/Al composite exhibits a very poor uniform compressive plastic strain (about 16.8%) and a very low ultimate compressive strength of 288.2 ± 10.0 MPa due to TiC and TiB_2_ agglomeration [28]. Conversely, both ultimate compressive strength and uniform compressive plastic strain first increase and then reduce with the increase in TiB_2_ and TiC content (Figure 7b). When the TiB_2_ and TiC content is 20 wt.%, the (TiC + TiB_2_)/Al composite demonstrates a high compressive yield strength of 196.4 ± 6.1 MPa and an ultimate compressive strength of 648.1 ± 20.1 MPa, as well as a good uniform compressive plastic strain of approximately 73.2%. As is well known, the compressive strength of Al matrix composites is strongly influenced by reinforcement content and the particle–matrix interface bonding strength. According to the literature [13], the α-Al grain size in (TiC + TiB_2_)/Al composites can be calculated by the Hall–Petch equation: $\Delta \sigma GR=k(d^{-\frac{1}{2}}-d_{m}^{-\frac{1}{2}})$. In this equation, k is the Hall–Petch parameter equal to 0.04 MPam^1/2^, and d and dm are the average grain size of the (TiC + TiB_2_)/Al composites and pure Al, respectively. According to the EBSD results in Figure 5, dm = 11.52 μm and d = 5.86, 5.76, 5.49, 2.02, 1.17, and 1.09 μm for the 5, 10, 15, 20, 25 and 30 wt.% (TiC + TiB_2_)/Al composites, respectively. The calculation results of Δσ are shown in Table 2. It can be seen that adding TiC and TiB_2_ particles can effectively improve the yield strength through grain refinement.

**Figure 8:**
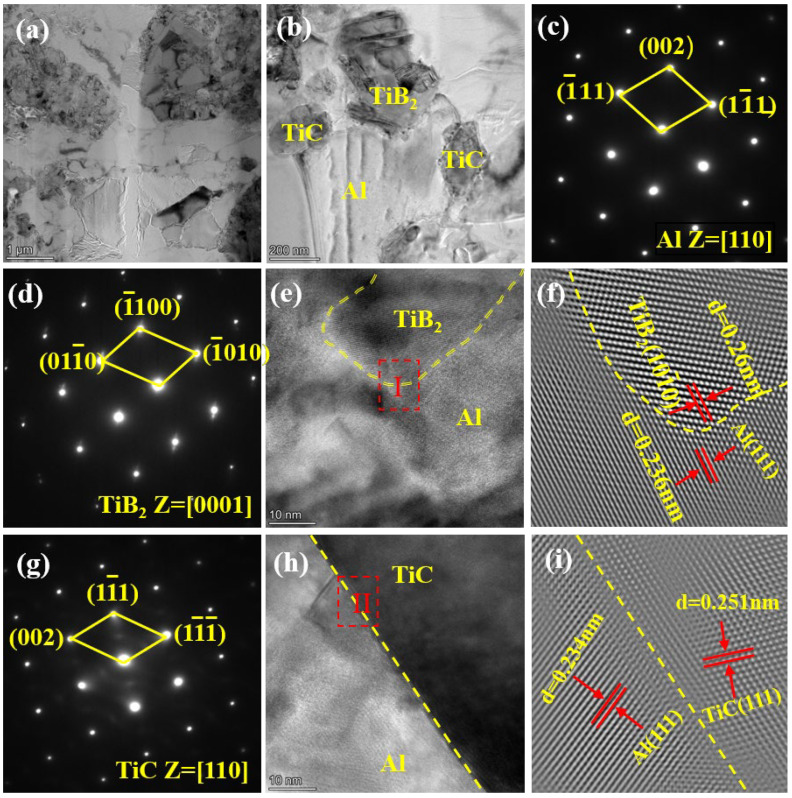
TEM micrographs of 20 wt.% (TiC + TiB_2_)/Al composite: (**a**,**b**) TEM images, (**c**) SAED of Al matrix, (**d**) SAED of TiB_2_, (**e**) HRTEM of TiB_2_/Al interface, (**f**) IFFT image of TiB_2_/Al interface of region I in (**e**), (**g**) SAED of TiC, (**h**) HRTEM of TiC/Al interface, and (**i**) IFFT image of TiC/Al interface of region II in (**h**).

Figure 8 shows the TEM images of the 20 wt.% (TiC + TiB_2_)/Al composite. It can be seen that TiB_2_ and TiC particles are uniformly distributed in the Al matrix. The TiB_2_ particles are irregular polygonal flakes with a hexagonal structure along the [001] zone axis, as confirmed by the SAED pattern in Figure 8d. The TiC particles are spherical-like shapes with a face-centred cubic (FCC) structure along the [110] zone axis, as confirmed by the SAED pattern in Figure 8g. The interface of TiB_2_/Al and TiC/Al is clean and straight, and there are no interface reactants as shown in Figure 8e,f,h,i. Furthermore, the inverse Fast Fourier transform (IFFT) pattern of region I indicates that the crystal planes of the Al matrix and the TiB_2_ particles have an orientation relationship of ${\left(011\right)}_{\mathrm{Al}}$//${\left(110\right)}_{\mathrm{Ti}B_{2}}$ and the lattice misfit is about 10.2%, regarded as a semi-coherent interface. The IFFT pattern of region II indicates that the crystal planes of the Al matrix and the TiC particles have an orientation relationship of ${\left(111\right)}_{\mathrm{Al}}$//${\left(111\right)}_{\mathrm{TiC}}$ and the lattice misfit is about 7.3%, regarded as a semi-coherent interface. The clean and strong interfacial bonding of Al/TiB_2_ and Al/TiC interfaces effectively transfers external loads from the Al matrix to the high-strength TiC and TiB_2_ particles and hinders crack propagation, thereby significantly enhancing the load-bearing capability [6,17,28,29]. Concurrently, it reduces the stress concentration at the Al/TiB_2_ and Al/TiC interfaces, which improves the strength and ductility and prevents premature failure [28,30]. Therefore, the synergy of grain refinement, dispersion strengthening and enhanced load-bearing capacity collectively accounts for the high strength and good plasticity in the (TiC + TiB_2_)/Al composites [6,17,28,29,30].

**Figure 9:**
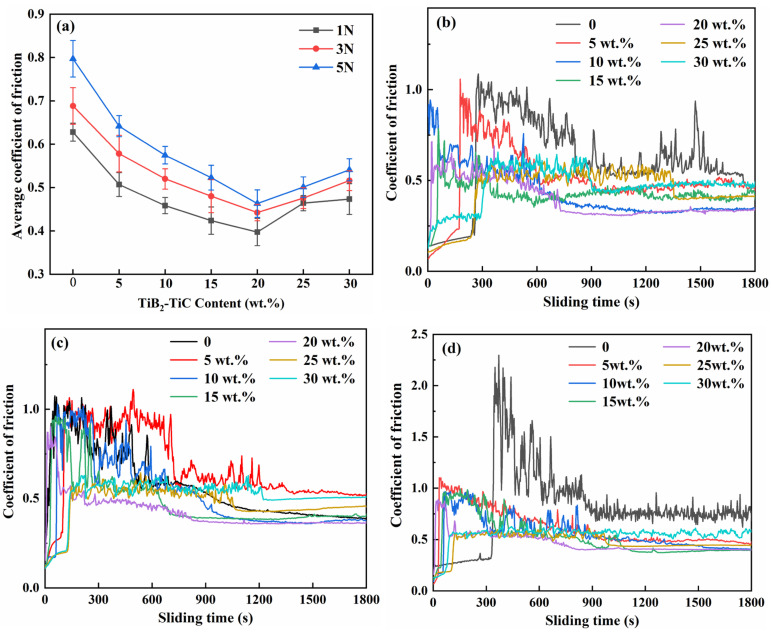
(**a**) The average coefficient of friction of the (TiC + TiB_2_)/Al composites and coefficient of friction curves as a function of time under different loads: (**b**) 1 N, (**c**) 3 N, and (**d**) 5 N.

Figure 9 presents the average COF and COF-versus-time curves of (TiC + TiB_2_)/Al composites with varying amounts of TiB_2_ and TiC contents. As shown in Figure 9a, the average COF of pure Al under applied loads of 1, 3 and 5 N is 0.604 ± 0.05, 0.678 ± 0.03 and 0.801 ± 0.04, respectively, showing much higher values than the (TiC + TiB_2_)/Al composites. At the same load, the average COF values of (TiC + TiB_2_)/Al composites first reduce and then increase with increasing TiB_2_ and TiC content. The (TiC + TiB_2_)/Al composites exhibit minimal COF values of 0.397 ± 0.03, 0.423 ± 0.03 and 0.467 ± 0.02 under applied loads of 1, 3 and 5 N, respectively, for the 20 wt.% (TiC + TiB_2_)/Al composites. However, for the 25 and 30 wt.% (TiC + TiB_2_)/Al composites, TiC and TiB_2_ agglomeration not only reduces the ductility of the Al matrix but also acts as abrasive debris during wear, leading to an increase in COF [28]. Moreover, the higher content of brittle phases also leads to severe abrasive wear, consequently leading to a high COF. In addition, the tribological properties vary significantly with varying TiC and TiB_2_ contents and loads (Figure 9b–d). Adding TiC and TiB_2_ particles can effectively reduce the run-in period. It can be seen that the run-in period is reduced from about 900, 600 and 900 s for pure Al to about 300, 150, 90 s for the 30 wt.% (TiC + TiB_2_)/Al composite. Moreover, compared to a low load, the high load not only introduces greater COF fluctuations but also accelerates the transition to a steady-state wear regime due to the increase in the contact zone between the specimen and abrasive and enhanced micro-ploughing efficiency [31].

**Figure 10:**
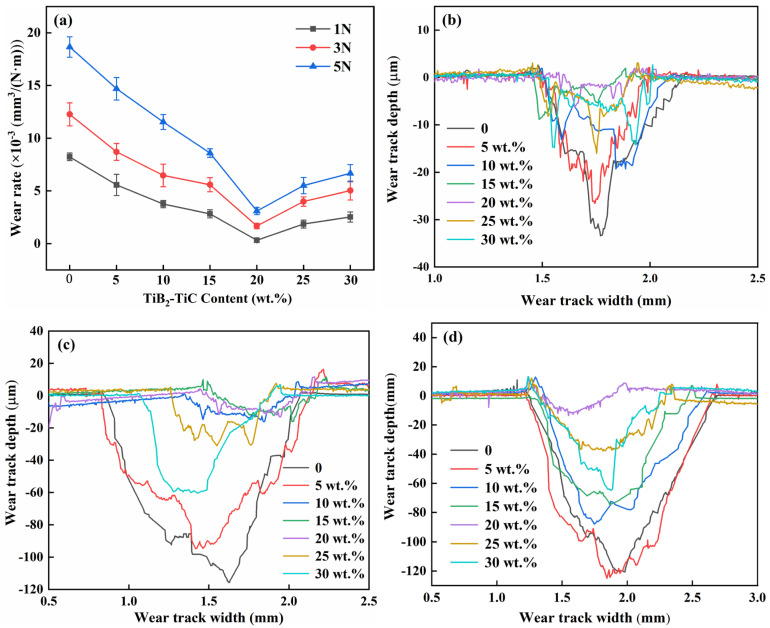
(**a**) The average wear rate of the (TiC + TiB_2_)/Al composites and two-dimensional wear diagram of the TiB_2_-TiC/Al composites under different loads: (**b**) 1 N, (**c**) 3 N, and (**d**) 5 N.

Figure 10 shows the average volumetric wear rates and two-dimensional wear scar profiles of the (TiC + TiB_2_)/Al composites. A marked rise in the wear rate is observed in (TiC + TiB_2_)/Al composites in Figure 10a with increasing applied load. Under the same test conditions, pure Al exhibits the highest wear rate at all loads, primarily due to the lower hardness and strength of pure Al compared to (TiC + TiB_2_)/Al composites. According to the Archard wear model (V ≈ K·P·s/H), wear volume is inversely related to material hardness [32]. The incorporation of TiB_2_ and TiC improves the strength of the Al matrix, thus improving the load-bearing capacity and consequently yielding excellent wear resistance [28]. Therefore, the wear scar depth and width are significantly reduced, leading to a substantially lower wear rate. When the content of TiB_2_ and TiC increases from 5 to 20 wt.%, the average volumetric wear rate significantly decreases to the minimum values of (3.143 ± 0.194) × 10^−4^ mm^3^/(N·m), 1.676 ± 0.251× 10^−3^ mm^3^/(N·m) and (3.093 ± 0.335) × 10^−3^ mm^3^/(N·m) at 1 N, 3 N, 5 N, respectively. These values are an order of magnitude lower than those for pure Al. However, further increasing the TiB_2_ and TiC content to 30 wt.% leads to a higher wear rate. This deterioration stems primarily from the agglomeration of TiC and TiB_2_ particles due to excessive addition, resulting in severe stress concentration and reduced ductility. This, in turn, promotes the initiation and propagation of cracks as well as brittle spallation during sliding, ultimately leading to a degradation in tribological performance [31]. Furthermore, excessive addition promotes the detachment of TiC and TiB_2_ during wear, which act as abrasive debris, inducing severe abrasive wear and thereby increasing the wear rate [28,31].

The 2D wear scar profiles of (TiC + TiB_2_)/Al composites under varying loads are displayed in Figure 10b–d. Pure Al exhibits the widest and deepest wear scar of about 658.8 and 33.4 μm, 1270. 9 and 115.6 μm, and 1472.5 and 120.6 μm at 1 N, 3 N and 5 N, respectively. The profile is uneven with significant fluctuations, indicating a relatively rough worn surface. In contrast, adding TiB_2_ and TiC particles can significantly reduce the scar width and depth and improve wear resistance. When the content of TiC and TiB_2_ is 20 wt.%, the smallest width and depth wear scars are about 474.9 and 6.2 μm, 740.2 and 11.4 μm, and 787.8 and 13.6 μm at 1 N, 3 N and 5 N, respectively, and minimal fluctuations are obtained in the (TiC + TiB_2_)/Al composites, showing excellent abrasive wear resistance. Conversely, when increasing the TiB_2_ and TiC content to 30 wt.%, both the wear scar width and depth (about 502.3/14.8 μm at 1 N, 891.5/60.3 μm at 3 N, and 1039.9/64.6 μm at 5 N) and wear rate (about 2.525 × 10^−3^, 5.033 × 10^−3^ and 6.674 × 10^−3^ mm^3^/(N·m) at 1 N, 3 N and 5 N, respectively) increase instead, as shown in Figure 10a. This is mainly because the excessive addition causes the agglomeration of TiC and TiB_2_ particles, which disrupts uniform stress distribution, reduces ductility, and provides abrasive debris during wear, thereby increasing scar size and wear rate [28,31,33,34]. Moreover, a higher TiC and TiB_2_ concentration elevates the surface hardness and roughness of the (TiC + TiB_2_)/Al composite, which promotes mechanical interlocking during the wear process, thereby leading to a higher COF and wear rate [35].

**Figure 11:**
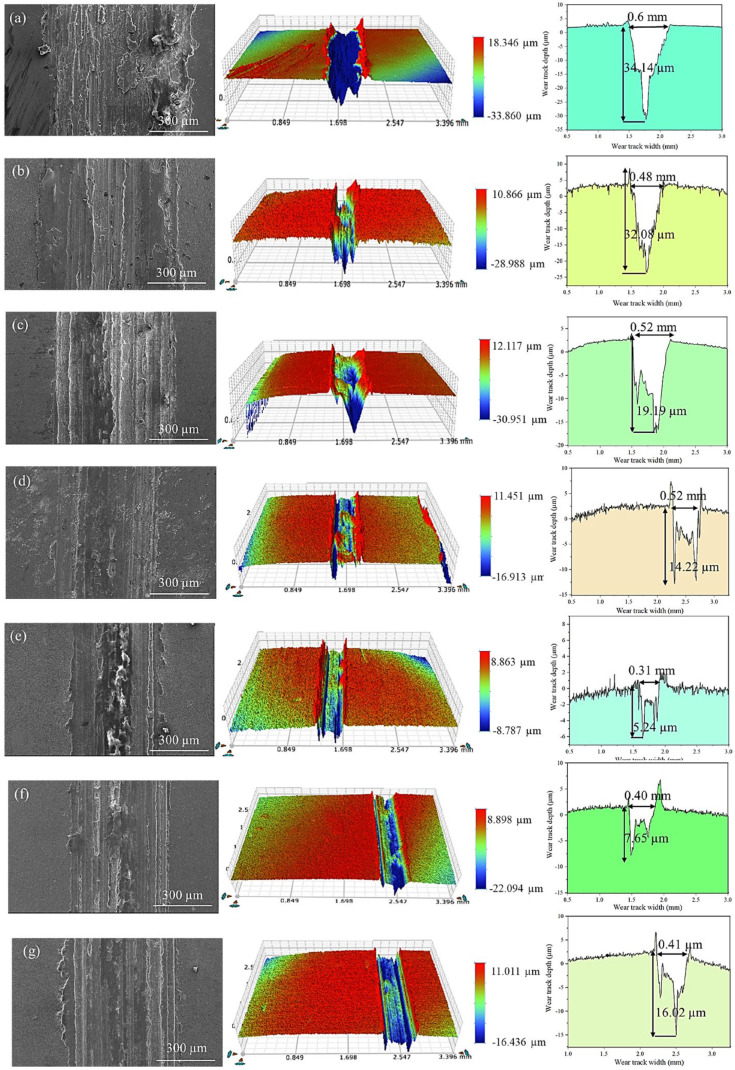
Wear surface morphology and profile of (TiC + TiB_2_)/Al composites with various contents of TiB_2_ and TiC at 1 N: (**a**) 0, (**b**) 5 wt.%, (**c**) 10 wt.%, (**d**) 15 wt.%, (**e**) 20 wt.%, (**f**) 25 wt.%, and (**g**) 30 wt.%.

To further reveal the wear mechanism of (TiC + TiB_2_)/Al composites, wear surface morphology and the associated performance of (TiC + TiB_2_)/Al composites under a 1 N load are characterized, as illustrated in Figure 11. For pure Al, the morphology exhibits distinct ploughing grooves and plastic deformation (Figure 11a), corresponding to the largest scar dimensions of 0.6 mm in width and 34.14 µm in depth. Compared with pure Al, the worn surfaces of the (TiC + TiB_2_)/Al composites exhibit progressively shallower ploughing grooves and reduced plastic deformation with the increase in TiB_2_ and TiC content from 5 to 20 wt.%, which is consistent with the gradual reduction in scar width and depth (Figure 11b–d). As shown in Figure 11e, the 20 wt.% (TiC + TiB_2_)/Al composite achieves the optimal performance, with a minimal wear scar of 0.31 mm in width and 5.24 µm in depth, demonstrating that an appropriate amount of TiB_2_ and TiC significantly enhances the wear resistance. The primary reason is that an optimal amount of TiB_2_ and TiC effectively enhances the hardness and strength of the Al matrix, thus improving its load-bearing capacity, reducing plastic deformation and suppressing both adhesive and abrasive wear, which collectively yield the best wear resistance performance [28,35]. In contrast, with further increases in TiB_2_ and TiC content, the wear scars become wider and deeper as shown in Figure 11f,g. The performance deterioration mainly stems from particle agglomeration and poor dispersion due to the excessive TiB_2_ and TiC content, which facilitates particle detachment during sliding and thereby degrades the tribological performance [36,37].

**Figure 12:**
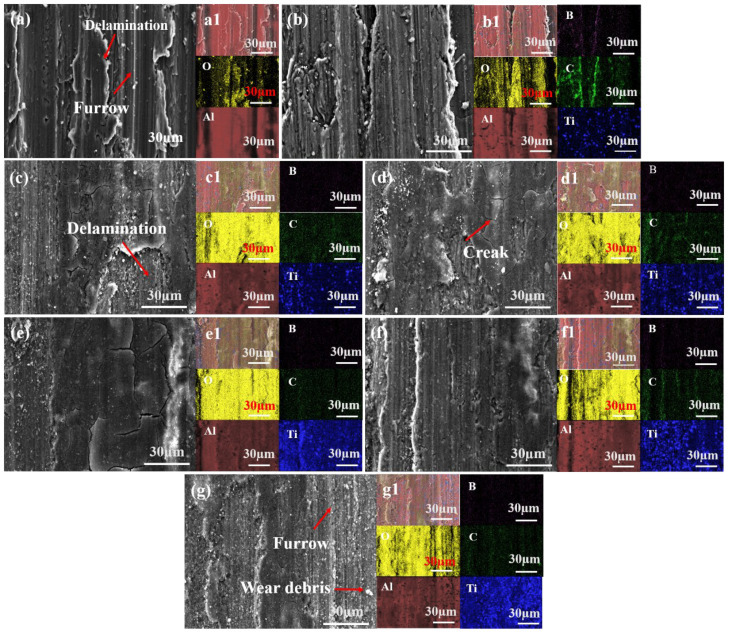
Micro-morphology and element distribution of the (TiC + TiB_2_)/Al composites with different contents of TiB_2_ and TiC after friction at 1 N: (**a**,**a1**) 0, (**b**,**b1**) 5 wt.%, (**c**,**c1**) 10 wt.%, (**d**,**d1**) 15 wt.%, (**e**,**e1**) 20 wt.%, (**f**,**f1**) 25 wt.%, and (**g**,**g1**) 30 wt.%.

Figure 12 shows the worn surface morphology and elemental distribution of (TiC + TiB_2_)/Al composites with different contents of TiB_2_ and TiC under a 1 N load. Severe adhesive wear, characterized by deep grooves and delamination, is evident in the pure Al alloy (Figure 12a). This is attributed to severe plastic deformation and the detachment of surface asperities, triggered by the normal load and shear stresses from the Al_2_O_3_ counterface. The EDS mapping of the wear track indicates that some oxide particles are formed in the detached Al debris due to the frictional heat as shown in Figure 12a. During the reciprocating sliding process, the generated oxide particles act as abrasive media and enhance the ploughing effect, resulting in deep grooves in the Al matrix. Moreover, the formation and spallation of the oxide layers occur cyclically due to the low support capacity of the soft Al matrix and repeated shear stress [28], thus leading to eventual delamination. Therefore, pure Al exhibits a higher COF and wear rate under severe adhesive wear.

The wear surface morphology of the 5 wt.% (TiC + TiB_2_)/Al composite (Figure 12b) shows little distinction from that of the pure Al matrix. However, as the TiB_2_ and TiC content increases to 10 and 15 wt.%, the wear surfaces of the (TiC + TiB_2_)/Al composite show a gradual reduction in both grooves and delamination regions as shown in Figure 12c,d. They exhibit a typical abrasive wear feature, characterized by shallow, parallel grooves aligned with the sliding direction [31]. According to the EDS mapping results as shown in Figure 12(c1,d1), there is uniform oxygen distribution across the worn surface, suggesting the formation of a continuous oxide-containing tribolayer. This layer effectively mitigates plastic deformation and tearing of the Al matrix, thereby resulting in a smoother tribo-oxidized film, fine wear debris, and shallower/narrower grooves as seen in Figure 12c,d. The EDS mapping results (Figure 12(c1,d1)) reveal that Ti, B and C elements are enriched in the debris, indicating the debris originates from TiB_2_ and TiC particles, which in turn improve the surface resistance to plastic deformation and abrasion, resulting in the dominant mechanism of abrasive wear [28]. Furthermore, the additional presence of numerous microcracks and spallation as well as obvious plastic deformation (Figure 12c,d) also implicates fatigue wear as a co-dominant mechanism on the worn surface. Thus, the primary wear mechanisms are a synergistic combination of abrasive and fatigue wear.

As shown in Figure 12e, the 20 wt.% (TiC + TiB_2_)/Al composite exhibits shallower ploughing grooves and less micro-cutting due to its enhanced load-bearing capacity, which results from its increased hardness and strength. Under applied stress, the fine wear debris is pressed into the worn surface, thereby forming a relatively continuous and smooth tribo-oxidized film composed of oxide matrix and ceramic particles. This harder tribo-oxidized film exhibits much stronger resistance to plastic deformation, abrasion and material spalling, thereby suppressing adhesive wear and shifting the dominant mechanism towards abrasion. Moreover, stress concentration often arises during reciprocating sliding, leading to the formation of microcracks within the film. The subsequent propagation of these cracks results in localized spallation, a characteristic of fatigue wear [38]. Furthermore, the harder tribo-oxidized film also minimizes direct contact between the Al matrix and Al_2_O_3_ counterface, thereby mitigating friction and wear and reducing the COF and wear rate in (TiC + TiB_2_)/Al composites.

Beyond 20 wt.% TiB_2_ and TiC, the (TiC + TiB_2_)/Al composite displays a worn surface characterized by abundant fine debris and elongated ploughing grooves (Figure 12f,g), indicating abrasive wear as the controlling mechanism. Although the high TiB_2_ and TiC content raises the hardness and strength of the (TiC + TiB_2_)/Al composite, it also induces a significant reduction in ductility due to the severe agglomeration of TiC and TiB_2_ particles. During reciprocating sliding, stress concentration facilitates crack initiation and propagation, culminating in the detachment of a large amount of hard debris under cyclic stress. Furthermore, the high-hardness TiB_2_ and TiC particles act as abrasives, cutting severely into the Al matrix and disrupting the continuity of the protective surface oxide film. This promotes severe abrasive wear and brittle spallation, which accounts for the higher COF and wear rate in (TiC + TiB_2_)/Al composites [39].

In summary, the dominant wear mechanism of (TiC + TiB_2_)/Al composites evolves with increasing ceramic content: from adhesive–abrasive wear to a mixed abrasive–fatigue wear, and finally to severe fatigue wear accompanied by brittle spallation. At low contents of TiC and TiB_2_ (≤10 wt.%), the Al matrix is directly exposed to the Al_2_O_3_ counterface and heavily penetrated due to the low load-bearing capacity, subsequently generating coarse debris and chips via deep grooving, leading to characteristic severe adhesive wear and abrasive wear. At an intermediate content (15–20 wt.%), uniformly dispersed TiC and TiB_2_ particles effectively improve the hardness and strength through grain refinement and dispersion strengthening, thereby improving load-bearing and abrasion resistance [40,41]. Moreover, the continuous oxide-containing tribolayer effectively increases the plastic deformation resistance and minimizes the direct contact between the Al matrix and Al_2_O_3_ counterface, thereby weakening cutting and adhesive wear [42,43]. Instead, abrasive wear and incipient fatigue wear occurs. However, beyond the optimal content (≥25 wt.%), the agglomeration of TiC and TiB_2_ particles causes severe stress concentration, leading to crack initiation and propagation and brittle spallation under cyclic stress. Moreover, the high-hardness TiB_2_ and TiC particles act as abrasives, leading to severe fatigue wear and brittle spallation. Overall, the 20 wt.% (TiC + TiB_2_)/Al composite exhibits optimal abrasive wear performance, which is ascribed to the synergistic effect of enhanced load-bearing capacity through grain refinement and dispersion strengthening and suppression of micro-cutting and adhesive wear through a continuous oxide-containing tribolayer.

## 4. Conclusions

In this work, micro/nano-scale (TiC + TiB_2_)/Al composites were successfully fabricated via Ti-B_4_C reactive sintering and SPS. The influence of TiB_2_ and TiC content on microstructure evolution, mechanical performance, and tribological properties was investigated. A reaction temperature of 1400 °C was confirmed to effectively synthesize the TiB_2_ and TiC ceramic particles. Adding moderate micro/nano-scale TiB_2_ and TiC particles (20 wt.%) significantly enhances the Vickers hardness, compressive yield strength, ultimate compressive strength and uniform compressive plastic strain, due to grain refinement and dispersion strengthening. Tribological test results indicate that the dominant wear behaviour shifts from adhesive and abrasive wear in pure Al to combined abrasion and fatigue wear in (TiC + TiB_2_)/Al composites. The 20 wt.% (TiC + TiB_2_)/Al composite exhibits superior wear resistance owing to a continuous tribo-oxidized film that effectively supports applied loads and suppresses matrix plastic deformation. Conversely, excessive addition of TiB_2_ and TiC deteriorates the wear resistance performance by promoting crack initiation and spallation through particle agglomeration, which in turn induces severe third-body abrasive wear and increases the COF and wear rate.

## Figures and Tables

**Table 1:** Relative density of (TiC + TiB_2_)/Al composites with varying TiB_2_ and TiC contents.

Alloys (TiC + TiB_2_ Content, wt.%)	0	5	10	15	20	25	30
Relative density (%)	99.4	99.2	99.1	99.2	99.2	99.1	98.9

**Table 2:** Contribution of grain refinement to yield strength of (TiC + TiB_2_)/Al composites.

Alloys (TiC + TiB_2_ Content, wt.%)	0	5	10	15	20	25	30
Δσ (MPa)	/	4.7	4. 9	5.3	16.4	25.2	26.5

## Data Availability

The original contributions presented in the study are included in the article. Further inquiries can be directed to the corresponding author.
